# Dairy cow personality: Consistency in a familiar testing environment

**DOI:** 10.3168/jdsc.2023-0499

**Published:** 2024-05-31

**Authors:** P. Hasenpusch, T. Wilder, A. Seidel, G. Thaller

**Affiliations:** Institute for Animal Breeding and Husbandry, Christian-Albrechts-University Kiel, 24098 Kiel, Germany

## Abstract

Personality affects the behavior of dairy cows in response to various situations on farm, such as novel or social challenges, which can affect the welfare of individual cows. Personality traits are evaluated through behavioral testing, typically in unfamiliar environments, but this can be a time-consuming process. Using a novel object test (NOT) and a forced human approach test (FHAT), we tested 222 individual dairy cows and retested a subset of cows (n = 78) 8 mo afterward. The aim was to see if personality traits can be identified in a familiar environment and if traits were consistent in between test and retest. Principal component analysis was used to identify personality traits. Extracted components (personality traits) were named according to determining variables (loadings <−0.63 or >0.63). Based on identified personality traits we calculated the intraclass correlation (ICC) coefficient and the differences between personality trait in test and retest to assess consistency. We identified 3 personality traits from NOT measurements (“explorative,” “boldness,” and “sociable”) explaining 85% of the variance and one trait from behavioral measurements in the FHAT (“trusting”) explaining 74% of the variance. Mean ICC of 0.6 with a standard deviation of 0.18 shows that although traits are consistent at herd level, cows react individually in stressful situations. As both tests were carried out in a familiar environment and results are similar to other studies that used unfamiliar environments, we can conclude that tests can be performed in a familiar environment.

## Highlights

• Personality traits can be extracted using NOT and FHAT in a familiar environment.
• Traits were stable in between test and retest, which were 8 months apart.
• Three traits were extracted from the NOT: explorative, bold, sociable.
• One other trait could be extracted from the FHAT: trusting.

## Graphical Abstract

**Figure:**
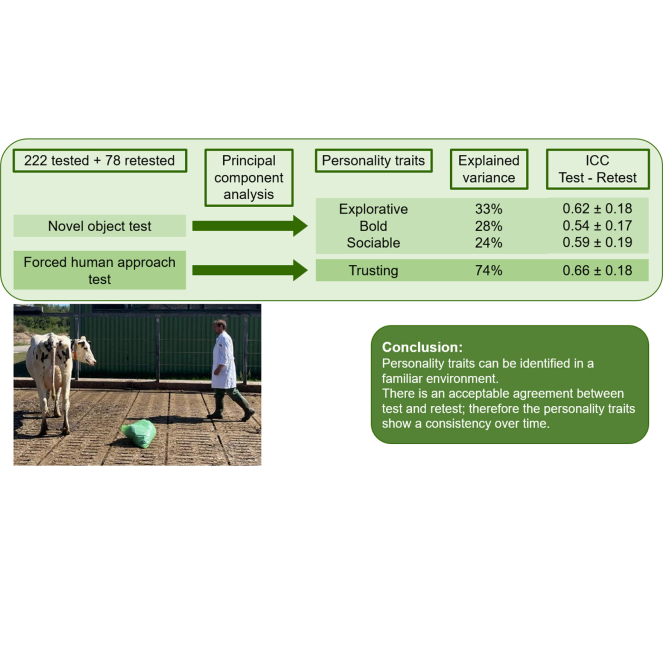
**Summary** Personality traits influence the welfare of dairy cows. Therefore, we tested 222 cows in a novel object test (NOT) directly followed by a forced human approach test (FHAT). Seventy-eight cows were retested 8 months afterward using the same tests, with the same object and approaching human. The aim was to see if personality traits can be extracted in a familiar environment and wherever these traits are consistent throughout time. The results of the principal component analysis revealed that 3 different personality traits, named explorative, bold, and sociable, could be extracted from the NOT (explaining 85% of the variance), and 1 other trait, trusting, could be extracted from the FHAT (explaining 74% of the variance). These traits proved to be consistent at the herd level with a variety of intraclass correlation (ICC) coefficients at the individual level.

Though dairy cow behavior is to some extent context specific, a growing body of literature suggests that their behavior is guided by certain underlying personality traits (Finkemeier et al., 2018; Foris et al., 2018; Neave et al., 2022). As the term personality varies in literature, we here refer to personality traits as “a correlated set of individual behavioral and physiological traits that are consistent over time and contexts” (Finkemeier et al., 2018, p. 3). The main factors of these traits are the cow's genes (Gutiérrez-Gil et al., 2008), her lifetime experience (Finkemeier et al., 2018), and the interdependencies between those 2 determinants. As there are many possible combinations and interactions of genetics and lifetime experiences, differences in personality may be very large within dairy herds. Knowledge about these differences is crucial because personality determines how a cow perceives her environment. This perception influences the experienced welfare and behavioral reaction to environmental stimuli (Dawkins, 1998). Thus, understanding individual personality traits carries insights to experienced welfare and optimizing potentials, highlighting the need for reliable personality trait validation. A reliable validation of personality entails that relationships between behavioral measurements and personality traits are consistent throughout tested individuals. Using behavioral tests, which confront animals with an unusual and stressful situation, interindividual consistency in behavioral reactions to these situations reflects personality traits. With this, the individual animal personality in relation to other animals can be inferred.

To determine whether personality traits can be identified in a familiar environment, we conducted a novel object test (**NOT**) and a forced human approach test (**FHAT**), which are well documented throughout literature (Forkman et al., 2007). These tests have the disadvantage that a lot of effort has to be put into standardization of testing conditions, such as constructing a testing arena (Foris et al., 2018) and cleaning the arena after every test (Foris et al., 2018), to make results comparable within cows of the same study. Consequently, the number of tested cows is limited, which in turn reduces the statistical power when analyzing the data with respect to differences in personality traits. Therefore, we focused on testing a high number of adult dairy cows to see if personality traits can be identified in a familiar environment. After 8 mo, a subset of animals was retested using the same procedure to confirm that identified traits qualify as personality traits that are consistent across time.

The tested dairy herd was housed at the research farm Karkendamm in the north of Germany, belonging to Kiel University. The lactating herd comprised 200 Holstein Frisian cows that were born and raised on the research farm. After entering the lactating herd, they stayed in 1 of 3 equally designed pens. Every pen was equipped with ad libitum access to water, 32 feeding bins that carry a mixed ratio of roughage and concentrates, and a cubicle:cow ratio of 1:1. All bins were emptied and refilled on a daily basis around 1000 h, resulting in ad libitum access to food. With 2 milking sessions a day (0600–0800 h and 1600–1800 h), tested cows generated 38 kg ± 8 kg milk per day, while being in their 2.2 ± 1.2 lactation with 186 ± 125 DIM.

In 3 different seasons (autumn 2022, winter 2022–2023, summer 2023), NOT followed by FHAT were conducted on 5 consecutive days. At each day of testing 2 groups of 10 cows were selected from the herd for testing, which sums up to 300 tested cows in total (3 testing seasons × 5 d of testing × 20 animals per day). All cows were eligible for testing the second day after estrus. The first group was selected at 0800 h and the second group was selected at 1100 h. Testing started 1 h after selection. During this hour cows were kept in a separate acclimatization pen. The aim of this procedure was to standardize the environmental conditions from which the cows emerged into the testing arena. Consistency of behavioral measurements was evaluated retesting all available animals (n = 78) from the first testing period in autumn 2022 using the same procedure, object, and human 8 mo later (±2.5 d).

Testing procedure started with gently leading the cow from the separate pen into the adjoining testing arena of 90 m^2^. This arena was familiar to the animals (part of the waiting area for the milking parlor) and pre-equipped with the novel object (a green plastic bag filled with metal containers). From entering the testing arena voluntarily, each NOT lasted 5 min. During the testing procedure cows had visual contact with conspecifics that were approximately 20 m away. None of the visible conspecifics were tested within the same season and cows under test did not take notice of them. In accordance with existing literature (van Reenen et al., 2004; Kilgour et al., 2006; MacKay et al., 2014; Foris et al., 2018), latency to approach and contact the object, latency to vocalize, number and duration of every contact, and number of vocalizations were scored by a trained and experienced observer. Latencies to contact or approach the object were measured from the cow's nose. A vocalization was characterized as any intended sound from the mouth, regardless of the volume. A wooden wall of 1.6 m covered the observer from toe to shoulder to ensure that tested animals were not affected by the observer. Due to practical reasons, we scored animals only live on a sheet of paper and did not make recordings of the behavioral tests. Latency and duration of behaviors were measured using a digital stopwatch. Based on the number and duration of every contact, the mean and SD of contact durations were calculated for every cow. Without removing cow or object from the arena, the FHAT started. An unknown human (male, 30 yr old, 185 cm tall) dressed in unfamiliar clothing (white gown) entered the testing arena and walked along the side until he reached the furthest corner from the animal (between 5 and 9 m from the animal). When the cow looked at the unknown human, the approach began in a speed of 1 step per second. During the whole approach, the distance between human and animal was continuously measured using a laser (DeWalt DW03050-XJ). Approach and measurement ended when the cow started moving any foot with the intention of moving any other direction than toward the human or there was a contact. Minimal distance between human and animal therefore is the last measurement obtained. Approaching from the furthest corner, relative to the animal, was repeated 3 consecutive times without leaving the arena. Minimal distances measured in all 3 approaches were used in the further analysis. Following the FHAT, the cow was led into her home pen and the next cow was brought into the arena for testing.

Due to tests being conducted in 3 different seasons and on 2 different parts of the day (morning and afternoon), climate and weather conditions before testing were very heterogeneous. Since this heterogeneity can affect measured behavioral parameters (Becker et al., 2020), we needed to correct for effects of weather. This was achieved by splitting the dataset into 6 parts (3 different seasons and 2 different parts of the testing day) and subtracting the mean value. Because parity can affect behavior as well (Davis et al., 2023), we corrected all variables by the mean value of the animals' assigned parity class. We assigned 3 parity classes in total, one to cows in their first, one to cows in their second, and one to cows being in their third or higher lactation. Additionally, we transformed left-sided variables by logarithmic transformation to reach normally distributed variables.

As the aim was to identify personality traits that systematically influence behavior, we could only use variables that shared variance with other variables in the further analysis. Therefore we calculated mean sampling adequacy (**MSA**), which is a measure of shared variance (Cerny and Kaiser, 1977). The MSA was calculated for NOT and FHAT variables separately. To subject variables to a principal component analysis (**PCA**), which we used to identify personality traits, literature suggests an MSA of 0.5 or higher (Budaev, 2010). Due to different contexts of applied behavioral tests (FHAT based on forced approach and the NOT based on the animal's will to approach), we deemed that separate PCA for each test would be appropriate. To avoid influences of recognition, we used behavioral measurements from cows that were exposed to NOT and FHAT for the first time as input to those PCA. To make interpretation easier, all components were rotated by the varimax rotation (Foris et al., 2018). According to the Kaiser rule, we only extracted components with an eigenvalue greater than 1 (Budaev, 2010). After extraction, components were named according determining variables, which had a loading of >0.63 or <−0.63. The extracted components were interpreted as personality traits. Within a component cows could be distinguished by a trait score, which is a numerical value reflecting the degree of personality trait expression (Equation 1; Varmuza and Filzmoser, 2009):

[1] $$Score_{ab}=\;\overset{n}{\underset{i=1}{\sum }}\;Loading_{bi}\times Measurement_{ai}.$$

Equation 1 shows that the trait score is a linear combination of variables (*i*) from an animal (*a*) and within a component (*b*). Loadings of variables represent the importance of a variable for a component.

To analyze the consistency of behavioral measurements, we calculated trait scores for the 78 cows we retested 8 mo later. We did this using Equation 1 filled with the loadings shown in Table 1, Table 2 and measurements of the retest. This procedure ensured that the same loadings were assigned to test and retest values, which allowed a direct comparison of traits scores between test and retest. Consistency of personality traits was analyzed using intraclass correlation (**ICC**), which was based on the personality trait scores calculated for every cow. In addition, the differences between personality trait scores of test and retest were calculated. Cows with a difference that was higher than herd mean ± 2 SD were labeled as cows with unusually high trait variation. For these cows, sickness data were checked to see whether illness could explain the variation.

**Table 1:** Principal components extracted from NOT: variable loadings, eigenvalues, and explained variance

Item	Component^1^		
	Explorative	Bold	Sociable
Measured variable			
Number of contacts	**0.83**	−0.24	−0.02
Mean duration of contact	**0.80**	−0.20	0.09
SD of contact duration	**0.94**	−0.13	0.00
Latency to approach the object	−0.21	**0.96**	0.04
Latency to contact the object	−0.27	**0.95**	0.08
Latency to vocalize	0.03	0.13	**0.90**
Number of vocalizations	−0.03	0.02	**−0.92**
Eigenvalue	2.31	1.94	1.67
Explained variance (%)	33	28	24

1 Numbers in bold font represent crucial loadings (<−0.63 or >0.63).

**Table 2:** Principal components extracted from FHAT: variable loadings, eigenvalues, and explained variance

Item	Trusting^1^
Measured variable	
Minimal distance 1. Approach	**0.85**
Minimal distance 2. Approach	**0.87**
Minimal distance 3. Approach	**0.87**
Eigenvalues	2.23
Explained variance (%)	74

1 Numbers in bold font represent crucial loadings (<−0.63 or >0.63).

Tested cows were in between 2 and 8.6 yr of age with a mean of 4 yr. Most of the cows were in their first (93 cows) or second (55 cows) lactation and the oldest was in her sixth lactation.

In 88% of the NOT, cows touched the object with a latency of 12.5 ± 54 s (median ± SD) and a mean contact duration of 2 ± 4.5 s. In the other 12% of tests, cows did not touch the object. In 93% of the tests, a cow vocalized after 31 ± 86 s (median ± SD), with 10 vocalizations per test on average. During the other 7% of NOT, cows did not vocalize. In case a cow did not touch the object or vocalize, associated latency variables were assigned the maximum possible latency (300 s) plus 1 s. On average the minimal approaching distance in the FHAT was 1.42 ± 0.82 m for the first approach, 1.35 ± 0.8 m for the second approach, and 1.31 ± 0.8 m for the third approach.

The MSA for the NOT was 0.58, with sampling adequacies ranging from 0.5 for vocalization to 0.67 for the number of contacts with the object. In the FHAT the MSA was 0.72, with sampling adequacies ranging from 0.71 for the last to 0.74 for the first approaching distance.

Using PCA we extracted 3 components from measured variables in the NOT (Table 1). The separate PCA conducted on measured variables in the FHAT revealed an additional component (Table 2). As shown, in Table 1, Table 2, 85% of the variance for the NOT and 74% of the variance in the FHAT were explained by the extracted components. The ICC also showed that consistency may differ at individual level. For being “explorative” the ICC was 0.62 on herd average with a SD of 0.18. For being “bold” (0.54 ± 0.17), being “sociable” (0.59 ± 0.19), and being “trusting” (0.66 ± 0.18), the results were quite similar. According to other studies (e.g., Czycholl et al., 2019), an ICC of 1 means perfect correlation, ICC between 1 and 0.7 is good, between 0.7 and 0.4 correlations are acceptable, and every correlation below 0.4 is unacceptable. Transferring these thresholds onto the results, we can conclude that consistency was acceptable on herd average. Additionally, the ICC shows that whereas for some cows the consistency was good, ICC values of other cows were classified as unacceptable.

The graphical analysis demonstrates that the differences between test and retest scores were normally distributed for every trait (Figure 1). This means that, for the majority of cows, personality trait scores of test and retest did not or just slightly differ. Compared with the NOT traits, differences in the FHAT trait were rather small. Based on a threshold of mean ± 2 SD, 5 cows with high variation in the trait “explorative” were identified. One of those differed substantially in her boldness score. No other cow differed vastly in her boldness score, 2 cows differed considerably in their sociability, and 3 cows differed in their “trust toward humans” scores, respectively. For these 8 cows, medical records were checked but there was no indication that could explain the high deviation.

**Figure 1:**
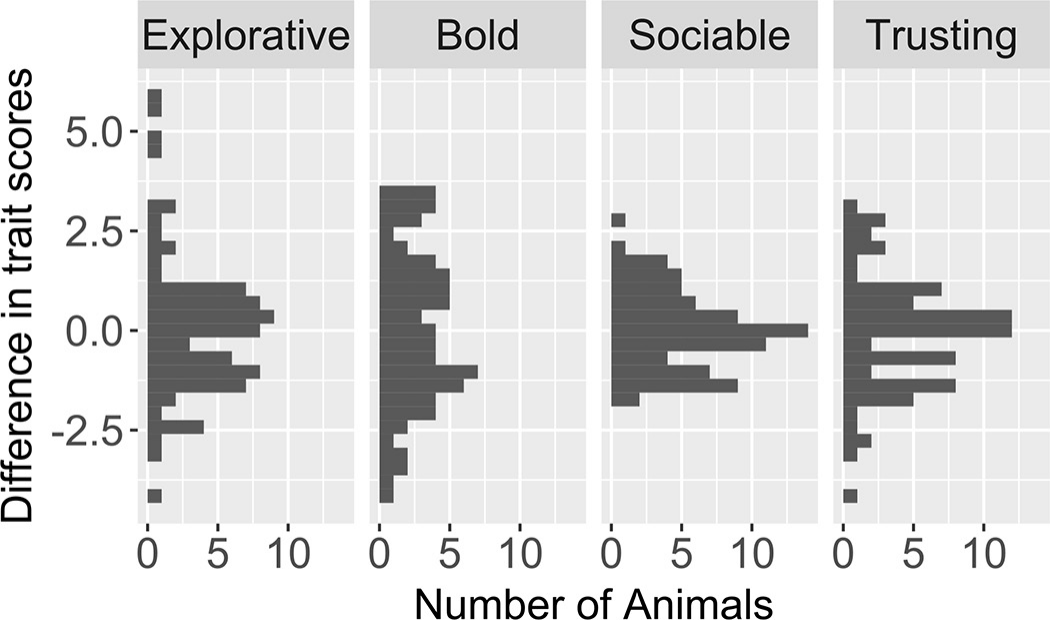
Differences between personality trait scores of test and retest for identified personality traits of 78 cows.

As shown in this study, personality traits of dairy cows can be identified in a familiar testing environment. A major benefit is that no time for acclimatization in a novel arena is needed (Foris et al., 2018). In practice more animals can be tested in the same amount of time, which can empower the feasibility of behavioral tests in farm animals. Additionally, the familiar environment is a practical solution for farms that do not have access to arenas specifically designed for behavioral tests. Since this holds for all commercial farms and links between animal personality and animal welfare have been established in previous studies (Dawkins, 1998; Réale et al., 2007; Richter and Hintze, 2019), our results may be of interest to welfare assessors for future assessments in commercial dairy farms. Moreover, the number of animals that can be tested in a particular timeframe increases, which yields advantages in conducting behavioral tests. A possible disadvantage is that lifetime experiences in the familiar environment may bias the results, which is an argument for the use of unfamiliar environments (Réale et al., 2010). Another reason for using an unfamiliar testing environment is that a study might focus on identifying personality traits related to reactions toward novel environments. This is especially relevant when cows are moved to a different stable or isolated from the herd for management purposes, highlighting why others have chosen such a setup. Although important variables differ, trait names in this and previous studies (MacKay et al., 2014; Foris et al., 2018) are similar; therefore, the effect of experiences in the familiar environment on behavioral responses can be neglected.

Another potential bias for results of behavioral tests is feed delivery and milking time in relation to the test (Jensen et al., 2023). However, inclusion of the test group effect in the statistical correction process should account for this potential bias. Additionally, cows had ad libitum access to food and water, which reduces the risk of stress due to hunger even more.

A limitation of this study is the lack of interobserver and intra-observer reliability calculations. This is because only one observer scored all animals live. This limits the application of the results to the observer in this study. However, the observer was experienced in the observation of behavioral tests from prior research and was trained beforehand to observe all tests included in this study. In this training, the correct application of the ethogram was compared with a second trained observer until an agreement was achieved. Therefore, the results of this study carry validity to some extent.

When contrasting the results of the FHAT to results of other studies regarding the human-animal relationship, sex and physical size of the unknown human have to be accounted for. In our case the approaching human was male and 1.85 m tall, which might be more intimidating than, for example, an approaching person of a smaller physique. Therefore, results of other FHAT may differ in their minimal distance between human and animal.

Comparing the results of this study with previous research that used the same tests, the highest variation of determining variables can be found for the explorative trait (MacKay et al., 2014; Foris et al., 2018). The explorative trait has therein been interpreted as walking or sniffing/licking the arena (or both). The different contexts in which the animal is explorative highlight that there are other aspects to this trait. As personality is defined “consistent across time and contexts” (Finkemeier et al., 2018), evaluation of contextual consistency is left for future studies.

Correlations of repeated measurements in other studies range from 0.29 to 0.63 (Foris et al., 2018; Neave et al., 2020). Since those correlations are based on Spearman rank correlations and we based our consistency on the ICC, the correlations are hardly comparable. The reason for using an opposing method is that the compared trait scores are normally distributed. This means that small changes in trait scores of a cow around the herd average score result in high changes of rank. As these small changes may be random variation, they may mistakenly lead to the conclusion that traits are not consistent. Therefore, using a variance-based approach such as ICC was deemed more appropriate.

Nevertheless, the high number of tested animals, which makes the results robust against a few cows with inconsistent trait scores, led to the assumption that the results of this study are comparatively consistent. Additionally, measurements of this study can be directly linked to the test performed, which is not possible in an unfamiliar environment because a novel arena test is executed at the same time.

The highest deviation between test and retest scores was observed in the trait “explorative.” Compared with the NOT, differences in the FHAT are rather small. One explanation might be that the human approach forces a reaction from the cow, whereas exploration, boldness, and being sociable in the NOT solely depend on the willingness to express the correlated behaviors.

That consistency at individual level can differ from the herd level was shown in Figure 1. Though most cows do not or just slightly differ between test and retest scores, some show distinct differences in 1 or 2 traits. Although medical records did not indicate any diagnosed disease, we cannot rule out an impact of discomfort that originated from recent agonistic interactions with herd members, or housing environment. To eliminate the risk of disturbances, which may cause inconsistencies, more test repetitions per cow would be necessary. As the NOT measures response to novel objects, recognition might be a problem with respect to interpretation when repeating this test. The same holds for the FHAT because former experiences with the approaching human may change the reaction of the cow (Waiblinger et al., 2002). Although there is no clear definition of minimal intervals for recognition to be negligible, results of previous studies regarding consistency of dairy cow behavior indicate that recognition might last up to a few weeks (Forkman et al., 2007). Since a longer interval between test and retest reduces the possibility of recognition even further, a conflict with the limiting factor age arises when stretching this interval. As one of the main goals of behavioral tests is to identify the personality of animals, which reflects the animal's general behavior in real situations, a better approach would be to permanently observe the animals in their home environment. In this regard, new technologies arise for automated behavioral tracking. Such systems could overcome the conflict between repeated testing and recognition and have the advantage that they are less labor intensive compared with executing behavioral tests. Moreover, the permanent observation provides a more holistic picture of certain behavior habits and includes the social interactions with herdmates in the home environment as a potential reason for discomfort.

We conclude that distinct personality traits can be reliably evaluated in a familiar environment. The major benefit is that more animals can be tested in a given time because there is no need for acclimatizing, which provides a higher statistical power of results. Although personality traits derived from measures of widely accepted behavioral tests explained most of the variance at the herd level, individual trait consistencies can differ to some extent. To gain higher certainty about trait consistency at individual level, repeated tests of an animal are needed. Though repeated testing is limited by age, automated behavioral tracking may solve this problem.
